# Systems Pharmacology Dissection of Traditional Chinese Medicine Wen-Dan Decoction for Treatment of Cardiovascular Diseases

**DOI:** 10.1155/2018/5170854

**Published:** 2018-05-10

**Authors:** Tao-Hua Lan, Lu-Lu Zhang, Yong-Hua Wang, Huan-Lin Wu, Dan-Ping Xu

**Affiliations:** ^1^Department of Cardiology, The Second Affiliated Hospital of Guangzhou University of Chinese Medicine, Guangzhou 510020, China; ^2^Center of Bioinformatics, College of Life Science, Northwest A&F University, Yangling, Shaanxi 712100, China; ^3^Beijing University of Chinese Medicine, Beijing 100029, China

## Abstract

Cardiovascular diseases (CVDs) have been recognized as first killer of human health. The underlying mechanisms of CVDs are extremely complicated and not fully revealed, leading to a challenge for CVDs treatment in modern medicine. Traditional Chinese medicine (TCM) characterized by multiple compounds and targets has shown its marked effects on CVDs therapy. However, system-level understanding of the molecular mechanisms is still ambiguous. In this study, a system pharmacology approach was developed to reveal the underlying molecular mechanisms of a clinically effective herb formula (Wen-Dan Decoction) in treating CVDs. 127 potential active compounds and their corresponding 283 direct targets were identified in Wen-Dan Decoction. The networks among active compounds, targets, and diseases were built to reveal the pharmacological mechanisms of Wen-Dan Decoction. A “CVDs pathway” consisted of several regulatory modules participating in therapeutic effects of Wen-Dan Decoction in CVDs. All the data demonstrates that Wen-Dan Decoction has multiscale beneficial activity in CVDs treatment, which provides a new way for uncovering the molecular mechanisms and new evidence for clinical application of Wen-Dan Decoction in cardiovascular disease.

## 1. Introduction

Cardiovascular diseases (CVDs) are the most common cause of death in the world [1]. Every year more than 10 million human lives are lost because of CVDs, and the mortality is predicted to increase to 23.6 million by 2030 [2]. The underlying mechanisms of CVDs are extremely complicated and not fully revealed, leading to a challenge for CVDs treatment in modern medicine. With the progress of modern medicine, various allopathic medicines with significant curative effects have been reported in recent years. However, there are still some medical problems of CVDs not solved satisfactorily with current western allopathic therapy.

Traditional Chinese medicine (TCM) characterized by multiple compounds and targets has shown its marked effects on various human diseases. Successful applications of Chinese herbal have been reported for CVDs prevention and treatment. Wen-Dan Decoction is one of the notable examples. Wen-Dan Decoction is composed of six herbs, including* Arum ternatum* Thunb,* Zingiber officinale* Roscoe, Caulis Bambusae in Taenia, Aurantii Fructus Immaturus,* Citrus reticulata*, and licorice. This decoction has been used to treat CVDs since Tang Dynasty, and the clinical effects have been validated in previous research. Clinical studies show that Wen-Dan Decoction achieved notable success in treating coronary heart disease [3], hypertension [4], arrhythmia [5], and heart failure [6]. Although the effect of Wen-Dan Decoction on CVD is promising, its pharmacological actions have not been fully revealed.

By integrating systems biology and pharmacology, systems pharmacology provides a new approach to reveal the complicated mechanisms of TCM in treating complicated diseases through pharmacokinetic evaluation, target prediction, and network/pathway analysis [7, 8]. Here, we developed a system pharmacology approach to explore the molecular mechanisms of Wen-Dan Decoction in CVDs treatment. First, we built a molecular database for all 6 herbs in Wen-Dan Decoction and used ADME system to screen the active compounds based on the above database. Next, potential targets were predicted and drug-target interactions were further constructed. Then, we constructed the networks to illustrate the molecular mechanisms of Wen-Dan Decoction in CVDs treatment. Finally, pathway integration analysis was performed to uncover CVDs pathway and therapeutic modules of target proteins. We believed that the results will significantly improve our understanding of the underlying mechanisms of Wen-Dan Decoction and provide new evidence for clinical application of Wen-Dan Decoction in cardiovascular disease.

## 2. Methods

**Figure 1:**
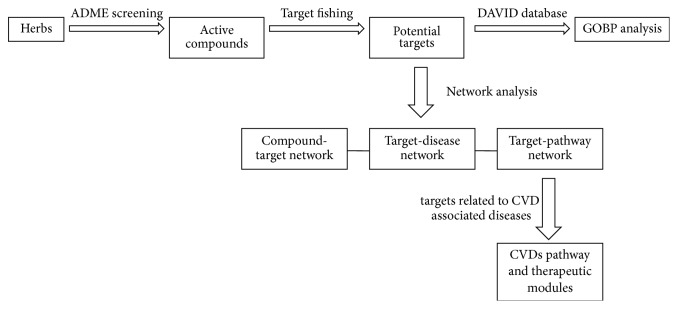
Systems pharmacology approach workflow.

The protocol of the systems pharmacology approach introduced in this work (Figure 1) includes 5 main steps as follows: (1) molecular database construction for all 6 herbs in Wen-Dan Decoction; (2) ADME evaluation to screen the active ingredients from the above compound database; (3) target-fishing to predict the direct targets of the obtained active compounds; (4) network construction and analysis to illustrate the molecular mechanism of Wen-Dan Decoction in treating CVDs; (5) pathway analysis to disclose CVDs pathway and therapeutic modules of target proteins.

### 2.1. Molecular Database Construction

A total of 140 active compounds of 6 drugs in Wen-Dan Decoction were manually collected from previously developed molecular database: Traditional Chinese Medicine Systems Pharmacology Database (TCMSP) [9].

### 2.2. ADME Evaluation

An in silico integrative model-ADME (absorption, distribution, metabolism, and excretion of drugs) including PreOB (predicts oral bioavailability) and PreDL (predicts drug-likeness) was used to screen the potential active compounds from Wen-Dan Decoction.

Considering that OB is one of the most crucial pharmacokinetic properties of orally administered drugs which has been proven to be efficient in the drug delivery to the systemic circulation, we introduced a robust in-house model OBioavail1.1 [10] to predict the OB value for drugs. The compounds with OB ≥30% were screened out for further analysis.

Considering that DL of molecules is one of the important factors in the ADME of human body, a PreDL model was developed to calculate the DL values of each active compound through evaluating the Tanimoto similarity [11] between compounds and chemicals in the Drugbank Database [12]. The compounds with DL ≥0.18 were screened out for further analysis.

### 2.3. Target-Fishing

Drug-targeting was firstly implemented by TCMSP Database and then an in-house model on the basis of ligand-target chemical genomics was introduced to enlarge the target library and increase the accuracy. We used Sys DT model which was developed on the basis of Random Forest (RF) and Support Vector Machine (SVM) algorithm to predict the target from potential active molecules of drugs. Only the targets with RF > 0.7 and SVM > 0.7 were reserved for further analysis.

To further explore functional annotation of the targets, a Gene Ontology Biological Process (GOBP) enrichment was performed through linking targets to DAVID [13] for classification. Only the terms with *P* value less than 0.05 were selected.

### 2.4. Network Construction

#### 2.4.1. Compound-Target (C-T) Network

In this section, a compound-target network was built to illustrate drug-target interactions of all active compounds in Wen-Dan Decoction and their potential targets. In this network, compounds are linked with their targeted proteins.

#### 2.4.2. Target-Disease (T-D) Network

To comprehensively understand the interrelationship between potential targets and diseases, a target-disease network linking target proteins with their relevant diseases and a target-CVDs (T-cD) network linking specific targets with CVDs were built by Cytoscape 2.8.1 [14], and the quantitative property “degree” of these networks was analyzed by Network Analysis plugin and CentiScaPe 1.2 of Cytoscape [15].

#### 2.4.3. Target-Pathway (T-P) Network

A Target-Pathway network is constructed by mapping the target proteins to the KEGG pathway database. The bipartite graphs were constructed by Cytoscape version 2.8.3. The compounds, targets, and pathways are represented by nodes, and the interaction between two nodes is represented by an edge. Node size is proportional to its degree.

#### 2.4.4. Pathway Constructions and Analysis

To explore the modulating specific pathways and the therapeutic feature of the active compounds on CVD treatment, pathways related to CVDs were picked out and assembled into a “CVDs pathway and therapeutic modules” under the pathological and clinical data.

## 3. Results

### 3.1. Active Compounds Identification

A total of 801 compounds were collected from the six herbs of Wen-Dan Decoction. As a result, 140 active compounds with OB ≥ 30% and DL ≥ 0.18 were obtained and 127 of 140 active compounds with drug targets were selected for further analysis (as displayed in Table S1). The top 5 molecules in degree ranking were presented in Table 1.

As shown in Table S1, there are 6 active compounds shared by two or more herbs of Wen-Dan Decoction. For instance, beta-sitosterol, a common ingredient of* Arum ternatum* Thunb and* Zingiber officinale* Roscoe, has inhibitory effects on the expression of VCAM-1 and ICAM-1, which promote atherosclerosis by regulating the chronic inflammatory process [16]. Stigmasterol, in* Arum ternatum* Thunb and* Zingiber officinale* Roscoe, has been found effective in inhibiting Ang II-stimulated vascular smooth muscle proliferation, in association with ROS reduction, SOD and CAT enhancement, and increase of p53 protein [17].

### 3.2. Drug-Targeting and Functional Analysis

2239 compound-target interactions were built between 127 compounds and 283 targets. The results showed that mostly compounds act on more than one target, and different compounds can have the same target. For instance, cavidine from* Arum ternatum* Thunb, stigmasterol, and beta-sitosterol from* Arum ternatum* Thunb and* Zingiber officinale* Roscoe, 6-methoxyaurapten from Aurantii Fructus, and medicarpin from licorice can interact with the same target CHRM3. Luteolin from Aurantii Fructus and quercetin from licorice can interact with the same target CDKN1A, which has effects on treatment of gastritis. Stigmasterol, beta-sitosterol, and coniferin from* Arum ternatum* Thunb and* Zingiber officinale* Roscoe and isosinensetin and sinensetin from Aurantii Fructus can interact with the same target ADRB2, which has effects on treatment of asthma.

**Figure 2:**
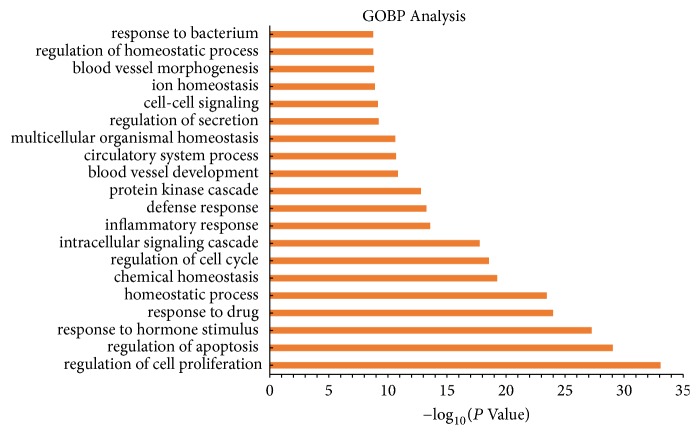
*Gene Ontology (GO) analysis*. The *y*-axis shows significantly enriched “biological process” categories in GO, and the *x*-axis shows the enrichment scores of those terms (*P* < 0.05).

In the subsequent GOBP enrichment analysis, we listed the top 20 significantly enriched GO terms (as displayed in Figure 2); the results showed that most of these targets are strongly correlated to inflammatory response, hormonal balance, and homeostasis, including response to drug, regulation of cell proliferation, blood vessel development, and multicellular organismal homeostasis.

### 3.3. Network Construction and Analysis

#### 3.3.1. Compound-Target (C-T) Network and Analysis

**Figure 3:**
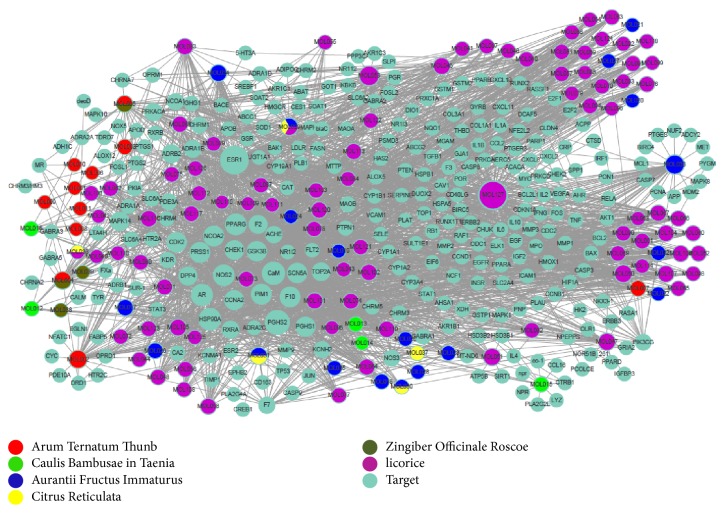
*C-T network*. Compounds are linked with their targeted proteins. Node size is proportional to its degree.

As shown in Figure 3, a compound-target interaction was generated based on 410 nodes (127 potential compounds and 283 potential targets) and 2239 edges. The average degree number of targets per compound is 18.055. The top 5 molecules and the top 5 targets in degree ranking were presented in Tables 1 and 2, respectively. Among those active compounds, MOL127 exhibits the highest degree (degree = 152), followed by MOL053 (degree = 63), MOL033 (degree = 56), and MOL050 (degree = 43), which indicated the multitarget properties of compounds. Among the candidate targets, ESR1 shows the highest degree (Degree = 141), followed by PGHS2 (degree = 103), CaM (degree = 86), and HSP90A (degree = 79), which demonstrated the potential therapeutic effect of Wen-Dan Decoction.

#### 3.3.2. Target-Disease (T-D) Network and Analysis

**Figure 4:**
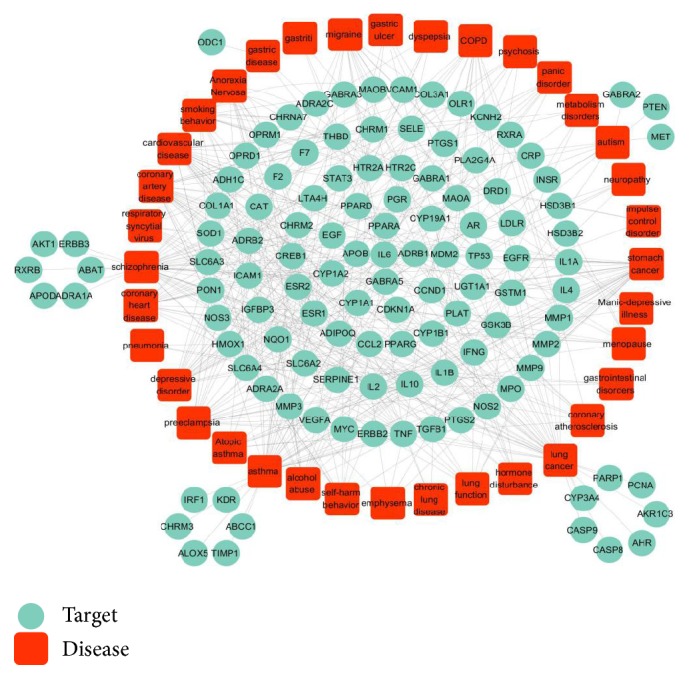
*T-D network*. Target proteins are linked with their correlated diseases and those diseases are linked with their correlated disease categories.

To comprehensively understand the interrelationship between potential targets and diseases, a target-disease interaction was built as shown in Figure 4. The results showed that multitargets are interrelated to the same diseases. For example, CDKN1A, IL1B, and TP53 are associated with gastritis; MPP1, MPP3, MPP9, PLAT, and SERPINE1 are associated with gastric ulcer; ADRA2A, SLC6A2, and SLC6A4 are associated with gastrointestinal disease. HTR2C, CYP1B1, CYP19A1, ESR1, ESR2, PDR, and AR are found to be interrelated to hormonal disorders; ABCC1, NQO1, TIMP1, ADRB2, and ALOX5 are associated with asthma. The results also revealed that multidiseases have the same target. For example, MPP3 and MPP9 are associated not only with gastric ulcer, but also with COPD, preeclampsia, and coronary artery diseases. SLC6A2 and SLC6A4 are associated not only with gastrointestinal disease, but also with anorexia nervosa, acute anxiety, and autism.

**Figure 5:**
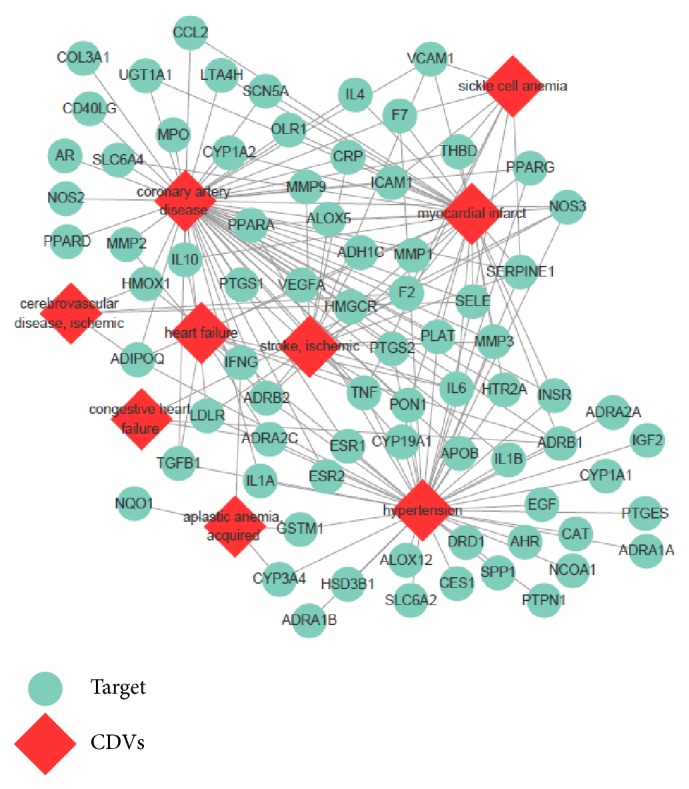
*Target-CVDs (T-cD) network*. Specific target proteins are linked with their correlated CVDs and CVDs are linked with their correlated disease categories.

To illustrate the interrelationship between specific targets and their correlated CVDs, a target-CVDs (T-cD) network was built as shown in Figure 5. The results showed that most targets related to more than one CVDs-associated disease and the shared targets might be the potential therapeutic targets in the treatment of CVDs. For example, NOS3, which plays an important role in regulation of nitric oxide production [18], is associated with coronary artery disease, myocardial infarct, congestive heart failure, stroke, and hypertension. As one of the highest incidences of CVDs, coronary artery disease links with more than 20 targets in this T-cD network, such as VCAM1 and ICAM1 (reported to increase in dysfunctional endothelial cells [19]), MMP9, MMP3, and MMP2 (being influencing factors in cardiac fibrosis [20]). Interleukin family was also predicted to be related to coronary artery disease, including IL 6, IL4, IL10, IL1A, and IL1B. All the results revealed the multitarget therapeutic efficiency of Wen-Dan Decoction in CVDs treatment.

#### 3.3.3. Target-Pathway (T-P) Network and Analysis

**Figure 6:**
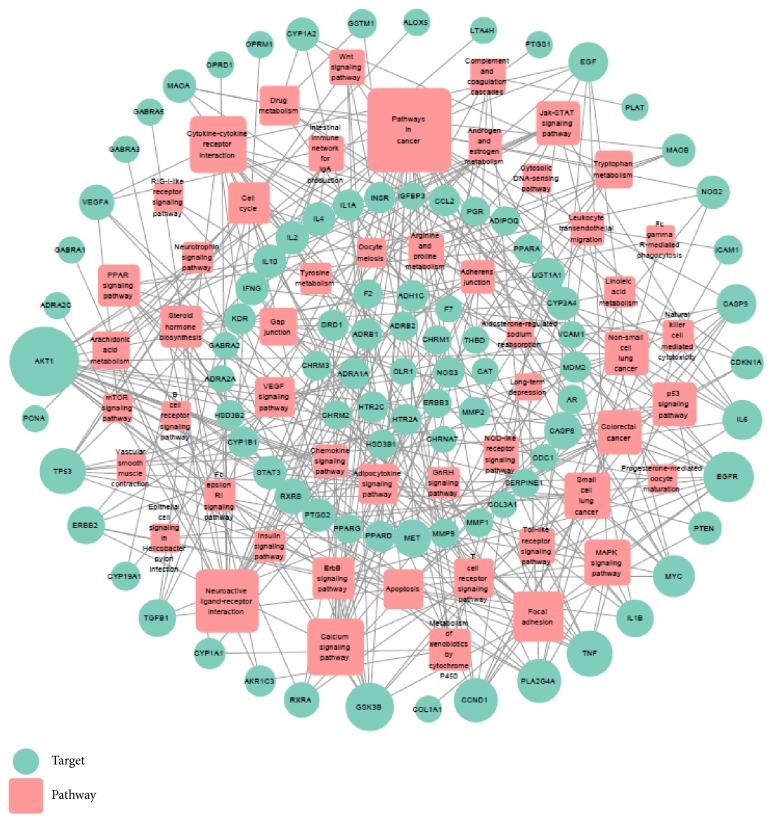
*T-P network*. The Target-Pathway network is constructed by mapping the target proteins to the KEGG pathway database. Node size is proportional to its degree.

As shown in Table 3, 92 targets are mapped to 51 pathways. A Target-Pathway interaction was built based on 143 nodes (92 potential targets and 51 potential pathways) and 324 edges (as shown in Figure 6). The results showed an average degree of 3.54 per target and 5.45 per pathway. Several target proteins (19/92) are mapped to more than 5 pathways, demonstrating that these targets may intercede the interactions between different pathways. The results also showed that the targets of Wen-Dan Decoction are mainly involved in the biological process of cancer, apoptosis, cell cycle, and so on. In addition, the main pathways coregulated by multiple targets, such as Ca^2+^ signal pathway and arachidonic acid metabolism pathway, have been validated as common pathways in the treatment of cardiovascular disease and stomach illness.

#### 3.3.4. Pathway Constructions and Analysis

**Figure 7:**
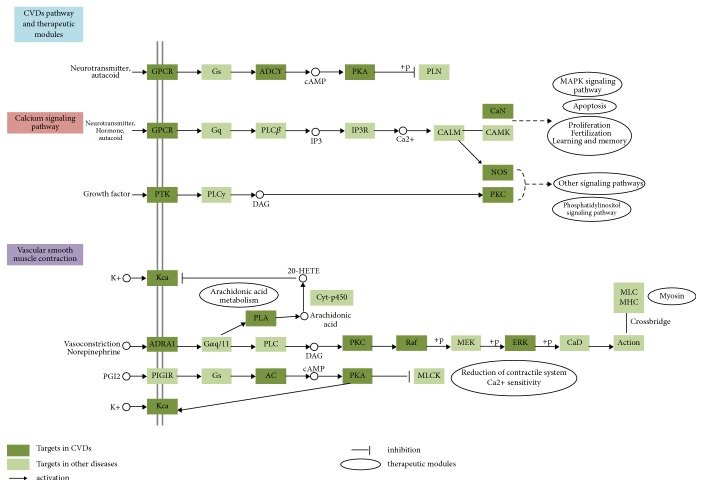
*CVDs pathway and therapeutic modules*. Distribution of target proteins of Wen-Dan Decoction on “CVDs pathway.” Arrows represent activation effect, and T-arrows represent inhibition effect.

In this section, a “CVDs pathway” was conducted based on the present cognition of CVDs pathology. As shown in Figure 7, this CVDs-associated pathway can be separated into two representative therapeutic modules (calcium signal pathway and vascular smooth muscle contraction), which reveal the underlying therapeutic effects of Wen-Dan Decoction.

Hypertension is one of independent risk factors for CVDs. The sustained high blood pressure may lead to the impairment of target organs, such as heart and kidney. Therefore, the therapies that can control blood pressure within the normal range are beneficial in the treatment of CVDs. As shown in Figure 7, therapeutic modules through the regulation of vascular smooth muscle contraction and calcium signal pathway are involved in the blood pressure regulation of Wen-Dan Decoction. For instance, norepinephrine signaling operates the function of vascular smooth muscle contraction through the regulation of some certain active compounds on their corresponding target proteins, including ADRA1, PKC, Raf, and ERK. Neurotransmitter GPCR and its downstream signal pathway are involved in the regulation of CAMK activity, which may cause cardiac hypertrophy and myocardial dysfunction [21]. All the results indicated that the blood pressure associate pathway is a potent therapeutic target of Wen-Dan Decoction in CVDs treatment.

## 4. Discussion

Cardiovascular disease remains the leading cause of human death around the world [22]. There are still some medical problems of CVDs not solved satisfactorily with current western allopathic therapy. The world is calling for a more efficient curative system. TCM is attracting more and more attention across the world for its marked effects in clinical practice. Wen-Dan Decoction, a clinically effective herb formula, has been used to treat CVDs specially accompanied by symptoms such as angina and arrhythmia. There is growing evidence showing that herbs and their active compounds in this decoction have biological effects on CVDs. For instance, Wen-Dan Decoction has been proven to regulate the disorder of lipid metabolism by raising the activity of total lipase (LA) and lipoprotein lipase (LPL) in the modal rats [23]. The elements of Wen-Dan Decoction and their active compounds, such as ginger [24, 25], Caulis Bambusae in Taenia [26], and licorice [27], also have been reported to attenuate the development of atherosclerotic lesions associated with a significant antihyperlipidemic effect. Besides, Wen-Dan Decoction can reverse hypertensive myocardial fibrosis and significantly reduce Ang II, ALDO in myocardial tissue, and plasma of SHR rats. The underlying mechanism may be related to the inhibition of the expression of TGF-beta 1, IGF 1, JNK, p38MAPK, and ERK5 in myocardial tissue [28, 29]. Citrus Reticulata and licorice exert cardioprotection by oxidative stress reduction, endogenous antioxidants augment, and structural integrity maintenance [30, 31]. However, the underlying mechanisms of action on the protein and pathway level are still unrevealed. Therefore, in this study, we developed a systems pharmacology approach to explore the mechanisms of Wen-Dan Decoction in CVDs treatment from a molecule to system level.

Previous studies suggested that TCM therapy has better influence on the complicated balance of whole cellular networks due to acting on multiple targets. The success examples of multitarget and combinatorial therapies indicated that systematic drug-design strategies should be directed against multiple targets [32]. In our work, a total of 801 compounds were collected from the six herbs of Wen-Dan Decoction. There are 6 active compounds shared by two or more herbs. 127 potential active compounds and their corresponding 283 direct targets were identified by ADME screening, demonstrating a multidrug-multitarget paradigm of Wen-Dan Decoction. Then, TCMSP Database and an in-house model on the basis of ligand-target chemical genomics were applied to conduct the drug-target interactions followed by GOBP analysis. 2239 compound-target interactions were found between 127 compounds and 283 targets. The analytical results distinctly revealed the action mode and biological processes that drugs utilized to achieve their curative effects. Finally, the networks were built for analysis to illustrate the molecular mechanism of Wen-Dan Decoction in CVDs treatment, and pathway analysis was performed to further dissect the therapeutic polypharmacology of Wen-Dan Decoction.

The underlying mechanisms of CVDs are extremely complicated and not fully revealed. We can tell from the results of this work that with multidrug-target-disease interactions active compounds of Wen-Dan Decoction achieve the curative results on CVDs treatment by regulating multiple targets and pathways. Since modern medicine is unable to prevent the medical failures and some ill-effects in CVDs treatment, TCM seems to be an alternative choice which shows great potential in confronting complex diseases.

## 5. Conclusion

In the present study, a systems pharmacology approach was developed by integrating the ADME screening, targets prediction, network, and pathway analysis to uncover the underlying mechanisms of Wen-Dan Decoction. Our results showed the following:

(1) 127 potential active compounds and their corresponding 283 direct targets were identified in Wen-Dan Decoction, demonstrating a multidrug-multitarget paradigm.

(2) In drug-targeting and functional analysis, 2239 compound-target interactions were found between 127 compounds and 283 targets, indicating that most compounds of Wen-Dan Decoction act on more than one target, and different compounds can have the same target.

(3) In network analysis, the C-T network indicated the multitarget properties of compounds, being the essence of the action mode of Wen-Dan Decoction. The T-D network showed that multitargets are interrelated to the same diseases and also revealed that multidiseases have the same target. The T-P network and CVDs pathway displayed that targets of Wen-Dan Decoction may intercede the interactions between different pathways, which further demonstrated the two therapeutic modules of Wen-Dan Decoction in CVDs treatment: calcium signal pathway and vascular smooth muscle contraction.

(4) This work provides a new approach for understanding the underlying mechanisms of Wen-Dan Decoction and new evidence for clinical application of Wen-Dan Decoction in cardiovascular disease.

## Figures and Tables

**Table 1:** Top 5 molecules in degree ranking.

MOL ID	Molecule name	Degree	Herb
MOL127	Quercetin	152	Licorice
MOL053	Kaempferol	63	Licorice
MOL033	Luteolin	56	Aurantii Fructus Immaturus
MOL050	7-Methoxy-2-methyl isoflavone	43	Licorice
MOL004	beta-Sitosterol	41	*Arum ternatum* Thunb & *Zingiber officinale* Roscoe

**Table 2:** Top 5 targets in degree ranking.

Target name	Degree
ESR1	141
PGHS2	103
CaM	86
HSP90A	79
AR	75

**Table 3:** Pathway and target interaction.

Pathway	Target
Pathways in cancer	E2F1, E2F2, PPARD, PTGS2, MMP9, PPARG, PTEN, MMP2, TGFB1, MMP1, AKT1, FOS, CASP3, CASP9, CASP8, NOS2, MYC, CHUK, PRKCA, EGFR, PIK3CG, AR, RXRB, RELA, RXRA, RUNX1T1, TP53, RB1, CDK2, MAPK1, CCND1, HIF1A, JUN, MAPK3, VEGFA, MDM2, MAPK8, ERBB2, EGLN1, BCL2L1, BCL2, NKX3-1, EGF, IL6, MET, RAF1, BIRC5, MAPK10, STAT1, STAT3, CDKN1A, GSK3B, RASSF1, BAX, IKBKB
Non-small cell lung cancer	PRKCA, E2F1, EGFR, PIK3CG, E2F2, RXRB, RXRA, ERBB2, TP53, RAF1, RB1, AKT1, MAPK1, CCND1, CASP9, RASSF1, MAPK3, EGF
Colorectal cancer	EGFR, PIK3CG, MET, TP53, RAF1, BIRC5, MAPK10, TGFB1, AKT1, MAPK1, FOS, CASP3, CCND1, CASP9, GSK3B, JUN, BAX, BCL2, MAPK3, MAPK8, MYC
Small cell lung cancer	E2F1, PIK3CG, E2F2, PTGS2, RXRB, RXRA, RELA, TP53, RB1, BCL2L1, PTEN, CDK2, AKT1, CCND1, CASP9, BCL2, NOS2, IKBKB, MYC, CHUK
Toll-like receptor signaling pathway	PIK3CG, IL6, TNF, RELA, MAPK10, CXCL11, STAT1, CXCL10, AKT1, MAPK1, FOS, MAPK14, JUN, MAPK3, CASP8, IL1B, MAPK8, IKBKB, CHUK, SPP1
VEGF signaling pathway	PRKCA, PIK3CG, PTGS2, RAF1, KDR, AKT1, MAPK1, PLA2G4A, CASP9, MAPK14, VEGFA, MAPK3, HSPB1, NOS3, PPP3CA, PLA2G2E, NFATC1
T cell receptor signaling pathway	IL4, PIK3CG, TNF, RELA, RAF1, IL10, AKT1, MAPK1, FOS, CD40LG, MAPK14, GSK3B, JUN, IFNG, MAPK3, PPP3CA, IKBKB, CHUK, NFATC1, IL2
Apoptosis	PIK3CG, TNF, RELA, TP53, BCL2L1, AKT1, CASP3, CASP9, BAX, CASP7, BCL2, CASP8, IL1B, PRKACA, PPP3CA, IKBKB, CHUK, IL1A
ErbB signaling pathway	PRKCA, EGFR, PIK3CG, ERBB3, ERBB2, RAF1, ELK1, MAPK10, AKT1, MAPK1, CDKN1A, GSK3B, JUN, MAPK3, MAPK8, EGF, MYC
Insulin signaling pathway	SREBF1, PIK3CG, HK2, ACACA, RAF1, ELK1, PDE3A, IGF2, MAPK10, AKT1, MAPK1, SLC2A4, PYGM, GSK3B, MAPK3, FASN, MAPK8, PRKACA, PTPN1, IKBKB, INSR
p53 signaling pathway	TP53, CHEK1, CHEK2, PTEN, CDK2, CCNB1, CDKN1A, CASP3, CCND1, CASP9, BAX, CASP8, SERPINE1, MDM2, IGFBP3
NOD-like receptor signaling pathway	IL6, CCL2, TNF, RELA, CXCL2, MAPK10, MAPK1, MAPK14, MAPK3, CASP8, IL1B, MAPK8, IKBKB, CHUK
Progesterone-mediated oocyte maturation	PIK3CG, ADCY2, RAF1, PDE3A, IGF2, MAPK10, CDK2, AKT1, CCNB1, PGR, MAPK1, MAPK14, MAPK3, PRKACA, MAPK8, CCNA2
MAPK signaling pathway	TNF, ELK1, TGFB1, AKT1, FOS, CASP3, IL1B, PRKACA, PPP3CA, EGF, MYC, CHUK, IL1A, RASA1, EGFR, PRKCA, RELA, TP53, RAF1, MAPK10, MAPK1, PLA2G4A, MAPK14, JUN, MAPK3, HSPB1, MAPK8, PLA2G2E, IKBKB
Focal adhesion	PRKCA, EGFR, PIK3CG, CAV1, ERBB2, MET, COL3A1, ELK1, RAF1, MAPK10, PTEN, KDR, AKT1, MAPK1, CCND1, GSK3B, JUN, BCL2, VEGFA, MAPK3, MAPK8, COL1A1, EGF, SPP1
Calcium signaling pathway	PRKCA, EGFR, PIK3CG, CAV1, ERBB2, MET, COL3A1, ELK1, RAF1, MAPK10, PTEN, KDR, AKT1, MAPK1, CCND1, GSK3B, JUN, BCL2, VEGFA, MAPK3, MAPK8, COL1A1, EGF, SPP1PRKCA, EGFR, DRD1, ADCY2, PTGER3, ERBB3, ERBB2, CHRM5, ADRB2, ADRB1, CHRM3, CHRM2, CHRM1, ADRA1B, ADRA1A, NOS3, CHRNA7, PRKACA, PPP3CA, NOS2, HTR2C, ADRA1D, HTR2A
GnRH signaling pathway	PRKCA, EGFR, ADCY2, RAF1, ELK1, MAPK10, MMP2, PRKCD, MAPK1, PLA2G4A, MAPK14, JUN, MAPK3, PRKACA, MAPK8, PLA2G2E
Fc epsilon RI signaling pathway	PRKCA, IL4, PIK3CG, TNF, RAF1, MAPK10, PRKCD, AKT1, MAPK1, PLA2G4A, MAPK14, MAPK3, MAPK8, PLA2G2E
Adipocytokine signaling pathway	PPARA, TNF, RXRB, RXRA, RELA, MAPK10, ADIPOQ, STAT3, AKT1, SLC2A4, MAPK8, IKBKB, CHUK
B cell receptor signaling pathway	PIK3CG, AKT1, MAPK1, FOS, RELA, JUN, GSK3B, MAPK3, RAF1, PPP3CA, IKBKB, CHUK, NFATC1
Steroid hormone biosynthesis	AKR1C3, CYP3A4, HSD3B2, CYP1B1, HSD3B1, CYP1A1, SULT1E1, UGT1A1, AKR1C1, CYP19A1
Neurotrophin signaling pathway	PIK3CG, RELA, TP53, RAF1, MAPK10, PRKCD, AKT1, MAPK1, MAPK14, GSK3B, JUN, BAX, BCL2, MAPK3, MAPK8, IKBKB
Cell cycle	E2F1, E2F2, TP53, CHEK1, RB1, CHEK2, TGFB1, CDK2, CCNB1, CCND1, CDKN1A, GSK3B, PCNA, MDM2, MYC, CCNA2
Chemokine signaling pathway	PIK3CG, ADCY2, CCL2, NCF1, RELA, CXCL2, RAF1, STAT1, CCL16, CXCL11, PRKCD, STAT3, CXCL10, AKT1, MAPK1, GSK3B, MAPK3, PRKACA, IKBKB, CHUK
Neuroactive ligand-receptor interaction	OPRM1, DRD1, GABRA2, GABRA1, PTGER3, GABRA3, GABRA5, PRSS1, CHRM5, ADRB2, ADRB1, CHRM4, CHRM3, GRIA2, CHRM2, CHRM1, F2, ADRA1B, ADRA2A, ADRA1A, ADRA2C, HTR2C, ADRA1D, HTR2A, OPRD1
Gap junction	PRKCA, EGFR, DRD1, ADCY2, GJA1, RAF1, MAPK1, ADRB1, MAPK3, PRKACA, EGF, HTR2C, HTR2A
Metabolism of xenobiotics by cytochrome P450	GSTM1, AKR1C3, CYP3A4, GSTM2, CYP1B1, CYP1A1, ADH1C, CYP1A2, UGT1A1, AKR1C1
Epithelial cell signaling in *Helicobacter pylori* infection	EGFR, CASP3, RELA, MAPK14, JUN, MET, MAPK8, MAPK10, IKBKB, CHUK
Vascular smooth muscle contraction	PRKCA, KCNMA1, MAPK1, PLA2G4A, ADCY2, MAPK3, ADRA1B, ADRA1A, RAF1, PRKACA, PLA2G2E, PRKCD, ADRA1D
Arachidonic acid metabolism	AKR1C3, PLA2G4A, PTGS2, PTGES, PTGS1, LTA4H, ALOX5, PLA2G2E, ALOX12
PPAR signaling pathway	PPARA, PPARD, OLR1, RXRB, RXRA, PPARG, ADIPOQ, MMP1, FABP5
RIG-I-like receptor signaling pathway	TNF, RELA, MAPK14, CASP8, MAPK8, MAPK10, IKBKB, CHUK, CXCL10
Cytokine-cytokine receptor interaction	EGFR, IL4, IL6, TNF, CCL2, MET, CXCL2, CCL16, CXCL11, IL10, TGFB1, CXCL10, KDR, CD40LG, VEGFA, IFNG, IL1B, EGF, IL1A, IL2
Drug metabolism	GSTM1, CYP3A4, GSTM2, MAOA, MAOB, ADH1C, CYP1A2, UGT1A1
Wnt signaling pathway	PRKCA, CCND1, PPARD, GSK3B, JUN, TP53, MAPK8, PRKACA, PPP3CA, MAPK10, MYC, FOSL1, NFATC1
mTOR signaling pathway	PIK3CG, AKT1, MAPK1, HIF1A, MAPK3, VEGFA, IGF2
Complement and coagulation cascades	PLAT, F10, THBD, F3, F2, SERPINE1, F7, PLAU
Jak-STAT signaling pathway	PIK3CG, IL4, AKT1, IL6, CCND1, IFNG, PIM1, BCL2L1, STAT1, MYC, IL10, STAT3, IL2
Tryptophan metabolism	CYP1B1, CYP1A1, MAOA, MAOB, CYP1A2, CAT
Aldosterone-regulated sodium reabsorption	PRKCA, PIK3CG, MAPK1, MAPK3, IGF2, INSR
Oocyte meiosis	CCNB1, PGR, MAPK1, AR, ADCY2, MAPK3, IGF2, PRKACA, PPP3CA, CDK2
Fc gamma R-mediated phagocytosis	PRKCA, PIK3CG, AKT1, MAPK1, PLA2G4A, NCF1, MAPK3, RAF1, PRKCD
Natural killer cell mediated cytotoxicity	PRKCA, PIK3CG, ICAM1, MAPK1, CASP3, TNF, MAPK3, IFNG, RAF1, PPP3CA, NFATC1
Intestinal immune network for IgA production	IL4, IL6, CD40LG, IL10, TGFB1, IL2
Long-term depression	PRKCA, MAPK1, PLA2G4A, GRIA2, MAPK3, RAF1, PLA2G2E
Androgen and estrogen metabolism	HSD3B2, HSD3B1, SULT1E1, UGT1A1, CYP19A1
Arginine and proline metabolism	ODC1, GOT1, MAOA, MAOB, NOS3, NOS2
Cytosolic DNA-sensing pathway	IL6, RELA, IL1B, IKBKB, CHUK, CXCL10
Adherens junction	EGFR, MAPK1, ERBB2, MET, MAPK3, PTPN1, INSR
Leukocyte transendothelial migration	PRKCA, PIK3CG, VCAM1, ICAM1, CLDN4, NCF1, MAPK14, MMP9, MMP2
Tyrosine metabolism	TYR, GOT1, MAOA, MAOB, ADH1C
Linoleic acid metabolism	CYP3A4, PLA2G4A, CYP1A2, PLA2G2E

## Supplementary Materials

Supplementary Materials Table S1: chemical information of the 127 active compounds which were collected from the six herbs of Wen-Dan Decoction, such as molecule name, OB(%), BBB, Caco-2, DL, degree, structure, and herb name.
