# Supplementary material for: Systems Pharmacology Dissection of Traditional Chinese Medicine Wen-Dan Decoction for Treatment of Cardiovascular Diseases
Source: Evid Based Complement Alternat Med. 2018 May 10;2018:5170854. doi: 10.1155/2018/5170854 (PMC5971304; doi:10.1155/2018/5170854)
Supplement: Supplementary Materials — Table S1: chemical information of the 127 active compounds which were collected from the six herbs of Wen-Dan Decoction, such as molecule name, OB(%), BBB, Caco-2, DL, degree, structure, and herb name. [file 5170854.f1.docx]

Table S1 Chemical information of 127 active compounds

| **ID** | **Molecule Name** | **OB (%)** | **BBB** | **Caco-2** | **DL** | **Degree** | **structure** | **Herb** |
| --- | --- | --- | --- | --- | --- | --- | --- | --- |
| MOL001 | 24-Ethylcholest-4-en-3-one | 36.08 | 1.22 | 1.46 | 0.76 | 2 | 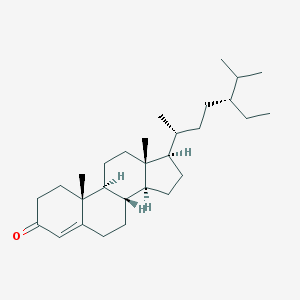 | Arum Ternatum Thunb |
| MOL002 | Cavidine1 | 35.64 | 0.63 | 1.08 | 0.81 | 28 | 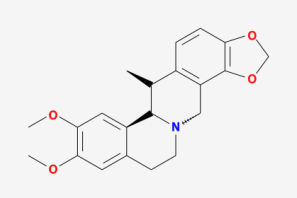 | Arum Ternatum Thunb |
| MOL003 | baicalein | 33.52 | -0.05 | 0.63 | 0.21 | 37 | 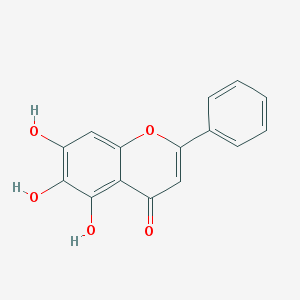 | Arum Ternatum Thunb |
| MOL004 | beta-sitosterol | 36.91 | 0.99 | 1.32 | 0.75 | 41 | 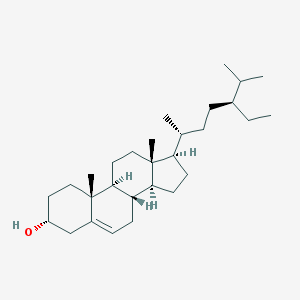 | Arum Ternatum Thunb＆Zingiber Officinale Roscoe |
| MOL005 | Stigmasterol | 43.83 | 1 | 1.44 | 0.76 | 31 | 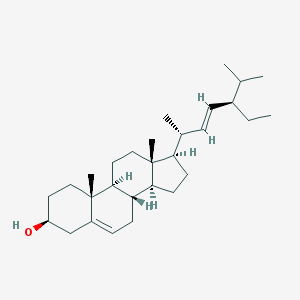 | Arum Ternatum Thunb＆Zingiber Officinale Roscoe |
| MOL006 | gondoic acid | 30.7 | 0.8 | 1.2 | 0.2 | 2 | 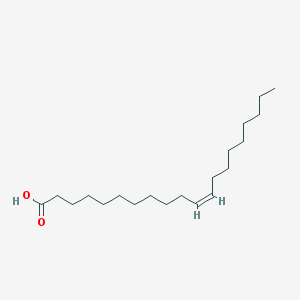 | Arum Ternatum Thunb |
| MOL007 | coniferin | 31.11 | -0.18 | 0.42 | 0.32 | 22 | 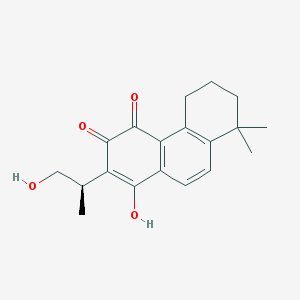 | Arum Ternatum Thunb |
| MOL008 | 10,13-eicosadienoic | 39.99 | 0.82 | 1.22 | 0.2 | 2 | 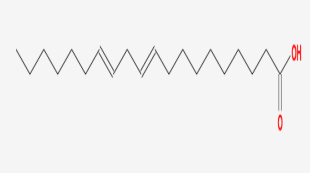 | Arum Ternatum Thunb |
| MOL009 | (3S,6S)-3-(benzyl)-6-(4-hydroxybenzyl)piperazine-2,5-quinone | 46.89 | -0.33 | 0.41 | 0.27 | 4 | 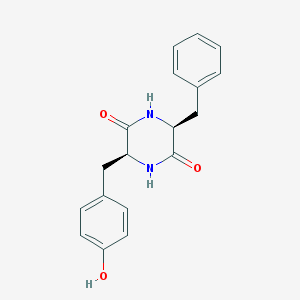 | Arum Ternatum Thunb |
| MOL010 | Cycloartenol | 38.69 | 1.33 | 1.53 | 0.78 | 1 | 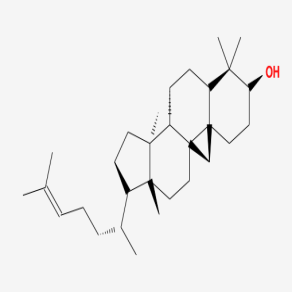 | Arum Ternatum Thunb |
| MOL011 | beta-D-Ribofuranoside, xanthine-9 | 44.72 | -1.84 | -1.21 | 0.21 | 3 | 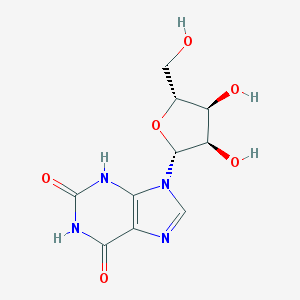 | Arum Ternatum Thunb |
| MOL012 | Syringaldehyde | 67.06 | 0.71 | 0.4 | 0.05 | 11 | 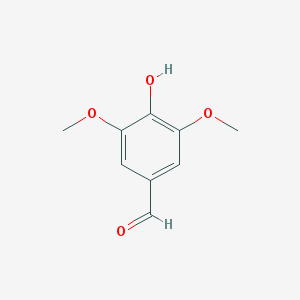 | Caulis Bambusae in Taenia |
| MOL013 | (+)-medioresinol | 87.19 | 0.5 | -0.29 | 0.62 | 11 | 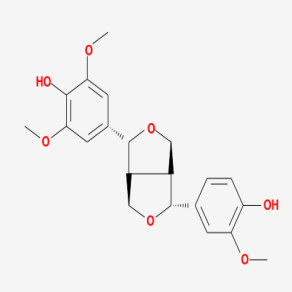 | Caulis Bambusae in Taenia |
| MOL014 | β-sitosterol | 33.94 | -0.44 | -1.37 | 0.7 | 1 | 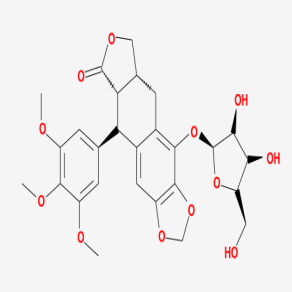 | Caulis Bambusae in Taenia |
| MOL015 | p-coumaric acid | 43.29 | 0.46 | 0.13 | 0.04 | 13 | 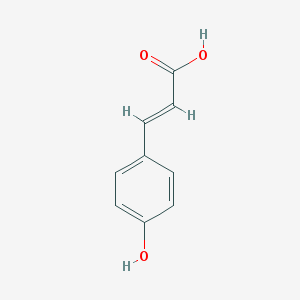 | Caulis Bambusae in Taenia |
| MOL016 | Ferulaldehyde | 49.26 | 0.82 | 0.58 | 0.05 | 10 | 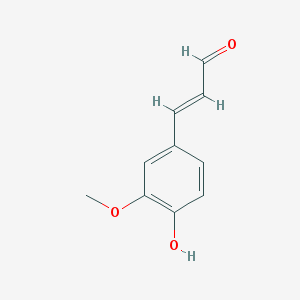 | Caulis Bambusae in Taenia |
| MOL017 | Isosinensetin | 51.15 | 0.03 | 1.16 | 0.44 | 27 | 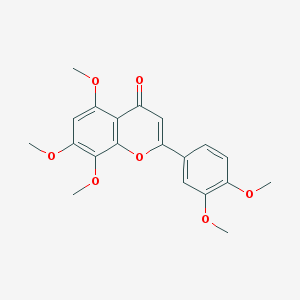 | Aurantii Fructus Immaturus |
| MOL018 | 5,7,4'-Trimethylapigenin | 39.83 | 0.12 | 1.01 | 0.3 | 16 | 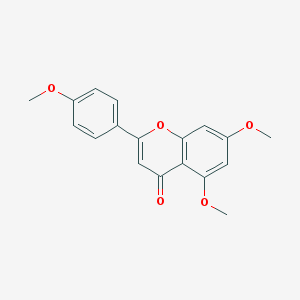 | Aurantii Fructus Immaturus |
| MOL019 | isosakuranetin-7-rutinoside | 41.24 | -2.38 | -1.59 | 0.72 | 1 | 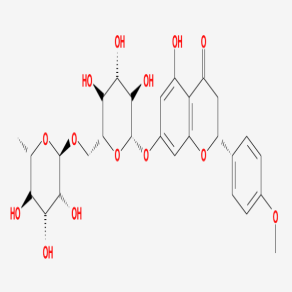 | Aurantii Fructus Immaturus |
| MOL020 | Prangenin | 43.6 | 0.29 | 0.8 | 0.29 | 3 | 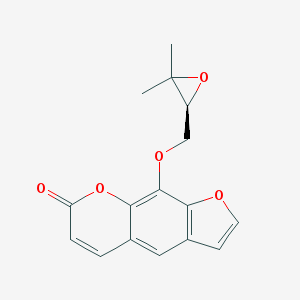 | Aurantii Fructus Immaturus |
| MOL021 | poncimarin | 63.62 | 0 | 0.66 | 0.35 | 4 | 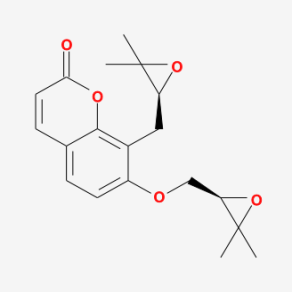 | Aurantii Fructus Immaturus |
| MOL022 | isoponcimarin | 63.28 | -0.02 | 0.5 | 0.31 | 4 | 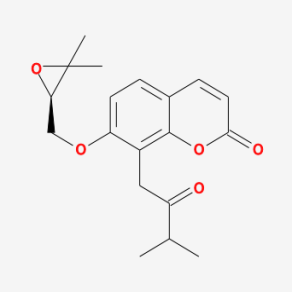 | Aurantii Fructus Immaturus |
| MOL023 | 6-Methoxy aurapten | 31.24 | 0.38 | 1.01 | 0.3 | 11 | 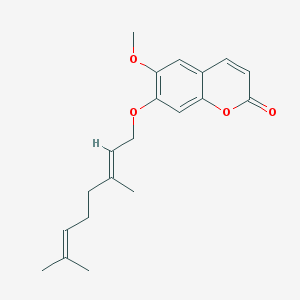 | Aurantii Fructus Immaturus |
| MOL024 | citrusin B | 40.8 | -3.22 | -1.94 | 0.71 | 1 | 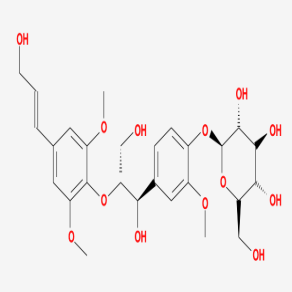 | Aurantii Fructus Immaturus |
| MOL025 | neohesperidin_qt | 71.17 | -0.47 | 0.26 | 0.27 | 7 | 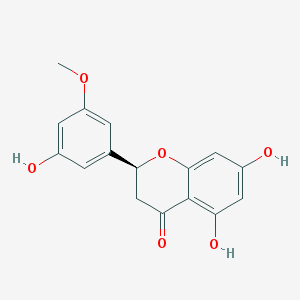 | Aurantii Fructus Immaturus |
| MOL026 | Sinensetin | 50.56 | 0.04 | 1.12 | 0.45 | 27 | 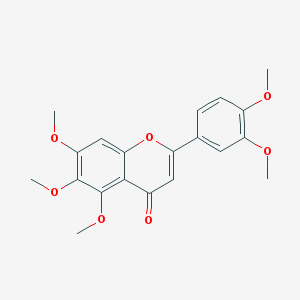 | Aurantii Fructus Immaturus |
| MOL027 | Ammidin | 34.55 | 0.92 | 1.13 | 0.22 | 8 | 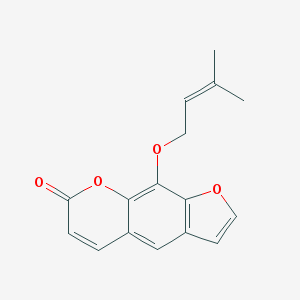 | Aurantii Fructus Immaturus |
| MOL028 | Eriodyctiol (flavanone) | 41.35 | -0.66 | 0.05 | 0.24 | 8 | 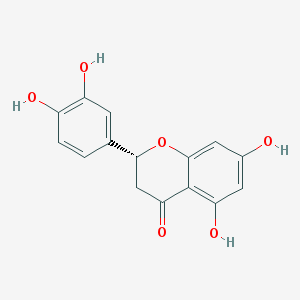 | Aurantii Fructus Immaturus |
| MOL029 | naringenin | 59.29 | -0.37 | 0.28 | 0.21 | 37 | 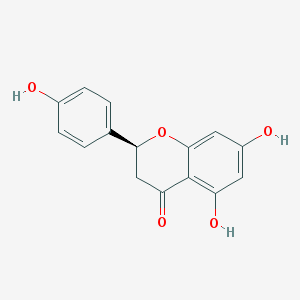 | Aurantii Fructus Immaturus＆Citrus Reticulata＆licorice |
| MOL030 | 5,7-dihydroxy-2-(3-hydroxy-4-methoxyphenyl)chroman-4-one | 47.74 | -0.3 | 0.28 | 0.27 | 10 | 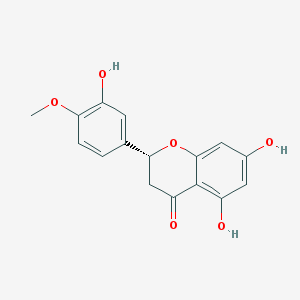 | Aurantii Fructus Immaturus＆Citrus Reticulata |
| MOL031 | nobiletin | 61.67 | -0.08 | 1.05 | 0.52 | 35 | 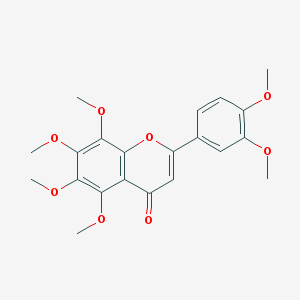 | Aurantii Fructus Immaturus＆Citrus Reticulata |
| MOL032 | didymin | 38.55 | -0.07 | 0.6 | 0.24 | 13 | 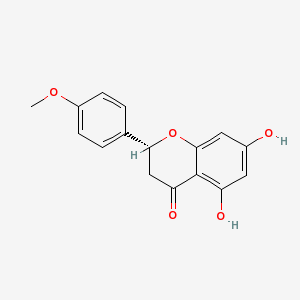 | Aurantii Fructus Immaturus |
| MOL033 | luteolin | 36.16 | -0.84 | 0.19 | 0.25 | 56 | 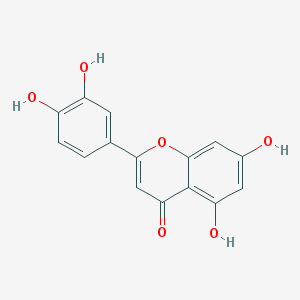 | Aurantii Fructus Immaturus |
| MOL034 | Tetramethoxyluteolin | 43.68 | 0.09 | 0.96 | 0.37 | 32 | 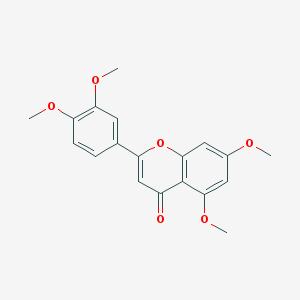 | Aurantii Fructus Immaturus |
| MOL035 | 4-[(2S,3R)-5-[(E)-3-hydroxyprop-1-enyl]-7-methoxy-3-methylol-2,3-dihydrobenzofuran-2-yl]-2-methoxy-phenol | 50.76 | -0.98 | 0.03 | 0.39 | 11 | 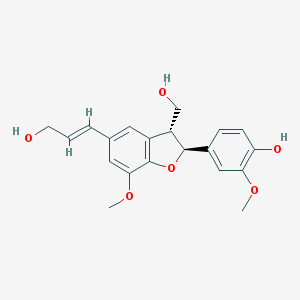 | Aurantii Fructus Immaturus |
| MOL036 | sitosterol | 36.91 | 0.87 | 1.32 | 0.75 | 3 | 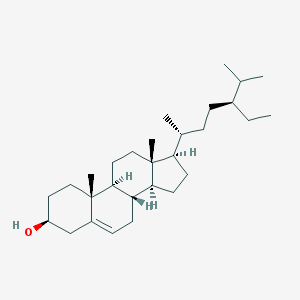 | Citrus Reticulata＆licorice |
| MOL037 | Citromitin | 86.9 | 0.16 | 0.88 | 0.51 | 10 | 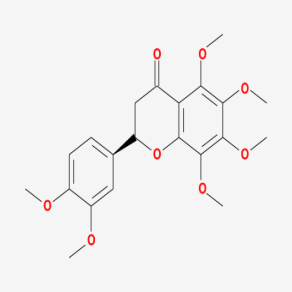 | Citrus Reticulata |
| MOL038 | 6-methylgingediacetate2 | 48.73 | -0.16 | 0.55 | 0.32 | 4 | 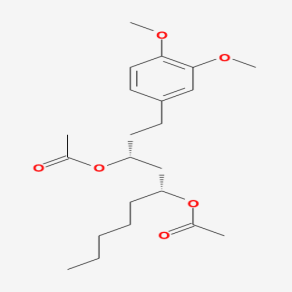 | Zingiber Officinale Roscoe |
| MOL039 | poriferast-5-en-3beta-ol | 36.91 | 1.14 | 1.45 | 0.75 | 2 | 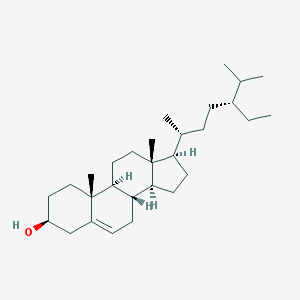 | Zingiber Officinale Roscoe |
| MOL040 | Inermine | 75.18 | 0.4 | 0.89 | 0.54 | 17 | 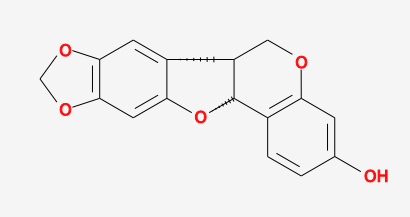 | licorice |
| MOL041 | Liquiritigenin | 32.76 | -0.29 | 0.51 | 0.18 | 12 | 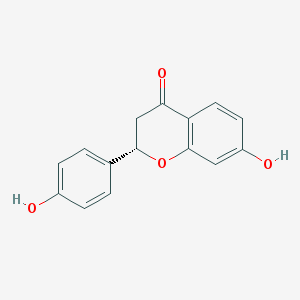 | licorice |
| MOL042 | Mairin | 55.38 | 0.22 | 0.73 | 0.78 | 1 | 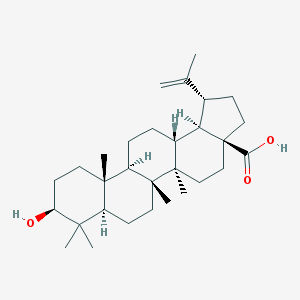 | licorice |
| MOL043 | Glycyrol | 90.78 | -0.2 | 0.71 | 0.67 | 11 | 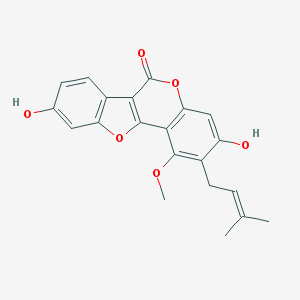 | licorice |
| MOL044 | Jaranol | 50.83 | -0.22 | 0.61 | 0.29 | 13 | 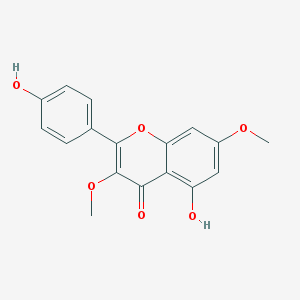 | licorice |
| MOL045 | Medicarpin | 49.22 | 0.53 | 1 | 0.34 | 34 | 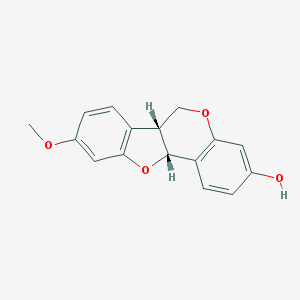 | licorice |
| MOL046 | Pinocembrin | 64.72 | 0.12 | 0.61 | 0.18 | 14 | 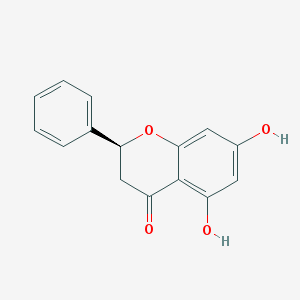 | licorice |
| MOL047 | isorhamnetin | 49.6 | -0.54 | 0.31 | 0.31 | 37 | 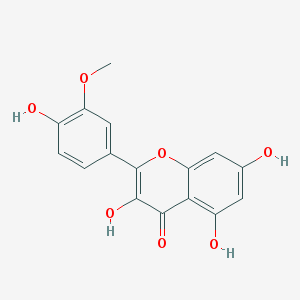 | licorice |
| MOL048 |  |  |  |  |  |  |  |  |
| MOL049 | sitosterol | 36.91 | 0.87 | 1.32 | 0.75 | 3 | 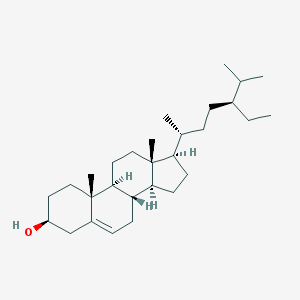 | licorice |
| MOL050 | 7-Methoxy-2-methyl isoflavone | 42.56 | 0.56 | 1.16 | 0.2 | 43 | 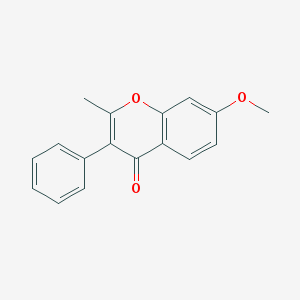 | licorice |
| MOL051 | formononetin | 69.67 | 0.02 | 0.78 | 0.21 | 39 | 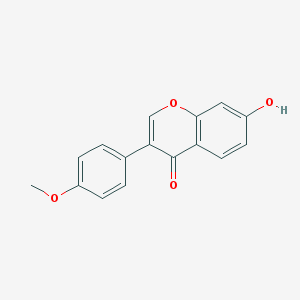 | licorice |
| MOL052 | Calycosin | 47.75 | -0.43 | 0.52 | 0.24 | 22 | 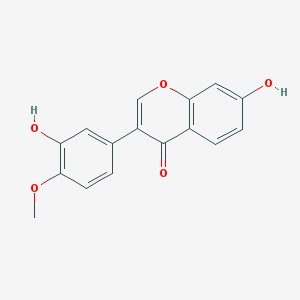 | licorice |
| MOL053 | kaempferol | 41.88 | -0.55 | 0.26 | 0.24 | 63 | 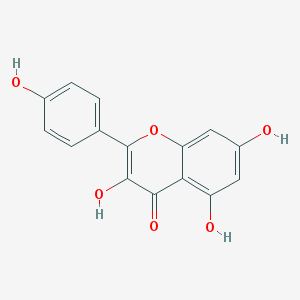 | licorice |
| MOL054 | (2S)-2-[4-hydroxy-3-(3-methylbut-2-enyl)phenyl]-8,8-dimethyl-2,3-dihydropyrano[2,3-f]chromen-4-one | 31.79 | 0.25 | 1 | 0.72 | 12 | 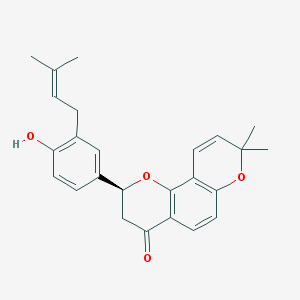 | licorice |
| MOL055 | euchrenone | 30.29 | 0.39 | 1.09 | 0.57 | 10 | 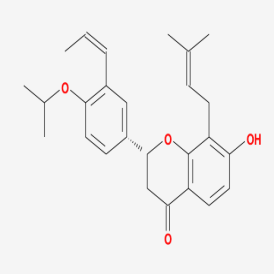 | licorice |
| MOL056 | glyasperin B | 65.22 | -0.09 | 0.47 | 0.44 | 21 | 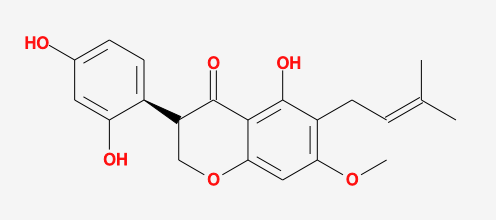 | licorice |
| MOL057 | glyasperin F | 75.84 | -0.15 | 0.43 | 0.54 | 18 | 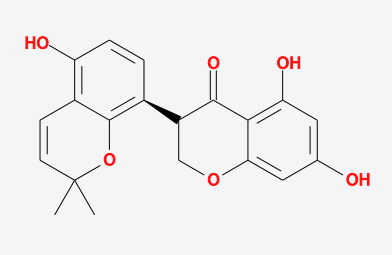 | licorice |
| MOL058 | Glyasperin C | 45.56 | 0.07 | 0.71 | 0.4 | 24 | 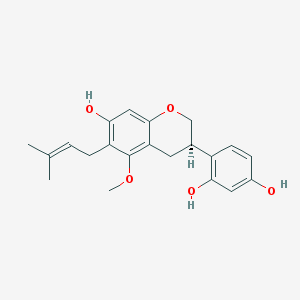 | licorice |
| MOL059 | Isotrifoliol | 31.94 | -0.25 | 0.53 | 0.42 | 14 | 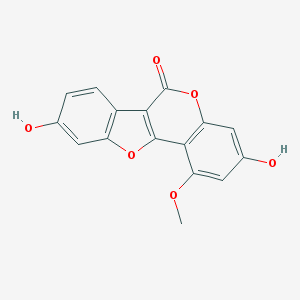 | licorice |
| MOL060 | (E)-1-(2,4-dihydroxyphenyl)-3-(2,2-dimethylchromen-6-yl)prop-2-en-1-one | 39.62 | -0.12 | 0.66 | 0.35 | 20 | 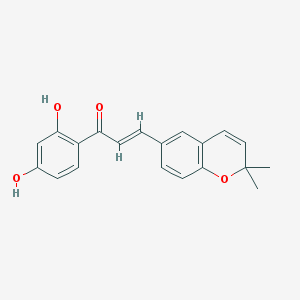 | licorice |
| MOL061 | kanzonols W | 50.48 | 0.04 | 0.63 | 0.52 | 21 | 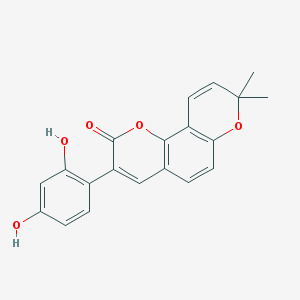 | licorice |
| MOL062 | (2S)-6-(2,4-dihydroxyphenyl)-2-(2-hydroxypropan-2-yl)-4-methoxy-2,3-dihydrofuro[3,2-g]chromen-7-one | 60.25 | -0.76 | 0 | 0.63 | 21 | 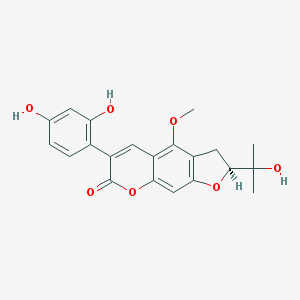 | licorice |
| MOL063 | Semilicoisoflavone B | 48.78 | -0.33 | 0.45 | 0.55 | 17 | 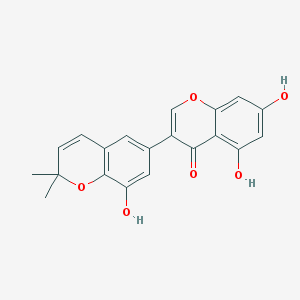 | licorice |
| MOL064 | Glepidotin A | 44.72 | 0.06 | 0.79 | 0.35 | 26 | 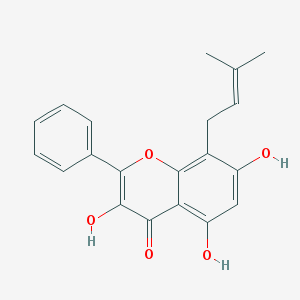 | licorice |
| MOL065 | Glepidotin B | 64.46 | -0.09 | 0.46 | 0.34 | 15 | 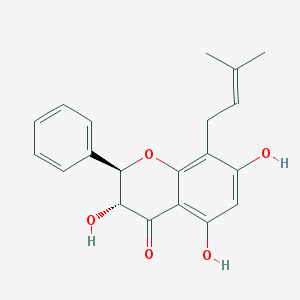 | licorice |
| MOL066 | Phaseolinisoflavan | 32.01 | 0.46 | 1.01 | 0.45 | 22 | 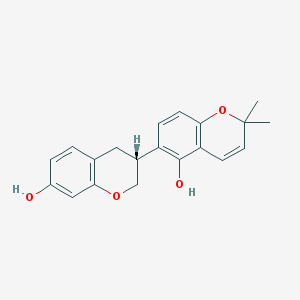 | licorice |
| MOL067 | Glypallichalcone | 61.6 | 0.23 | 0.76 | 0.19 | 27 | 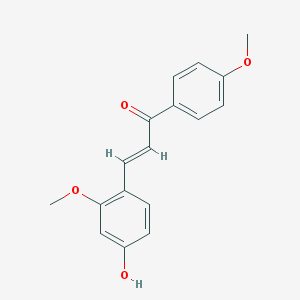 | licorice |
| MOL068 | 8-(6-hydroxy-2-benzofuranyl)-2,2-dimethyl-5-chromenol | 58.44 | 0.34 | 1 | 0.38 | 6 | 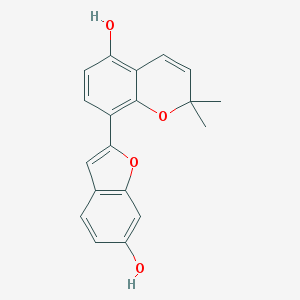 | licorice |
| MOL069 | Licochalcone B | 76.76 | -0.46 | 0.47 | 0.19 | 19 | 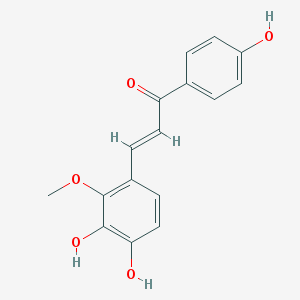 | licorice |
| MOL070 | licochalcone G | 49.25 | -0.04 | 0.64 | 0.32 | 17 | 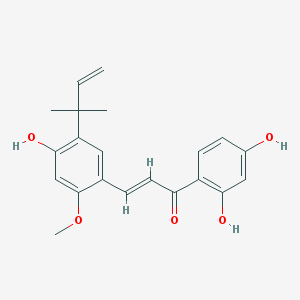 | licorice |
| MOL071 | 3-(2,4-dihydroxyphenyl)-8-(1,1-dimethylprop-2-enyl)-7-hydroxy-5-methoxy-coumarin | 59.62 | -0.23 | 0.4 | 0.43 | 23 | 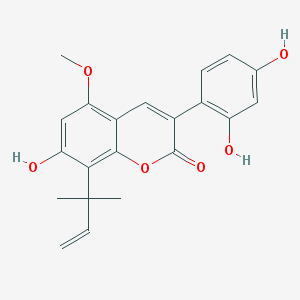 | licorice |
| MOL072 | Licoricone | 63.58 | -0.14 | 0.53 | 0.47 | 15 | 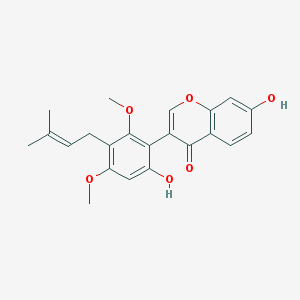 | licorice |
| MOL073 | Gancaonin A | 51.08 | 0.13 | 0.8 | 0.4 | 20 | 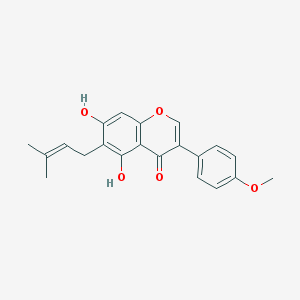 | licorice |
| MOL074 | Gancaonin B | 48.79 | -0.1 | 0.58 | 0.45 | 22 | 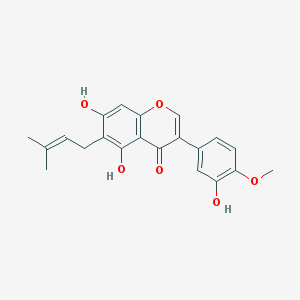 | licorice |
| MOL075 | 3-(3,4-dihydroxyphenyl)-5,7-dihydroxy-8-(3-methylbut-2-enyl)chromone | 66.37 | -0.13 | 0.52 | 0.41 | 18 | 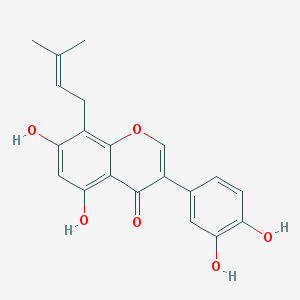 | licorice |
| MOL076 | 5,7-dihydroxy-3-(4-methoxyphenyl)-8-(3-methylbut-2-enyl)chromone | 30.49 | 0.21 | 0.9 | 0.41 | 20 | 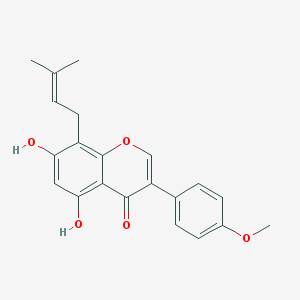 | licorice |
| MOL077 | 2-(3,4-dihydroxyphenyl)-5,7-dihydroxy-6-(3-methylbut-2-enyl)chromone | 44.15 | -0.28 | 0.48 | 0.41 | 16 | 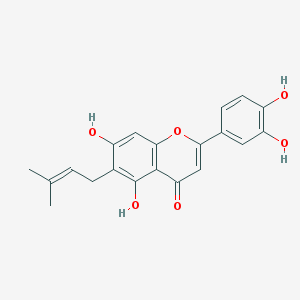 | licorice |
| MOL078 | Glycyrin | 52.61 | -0.13 | 0.59 | 0.47 | 17 | 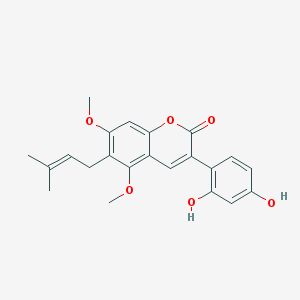 | licorice |
| MOL079 | Licocoumarone | 33.21 | 0.06 | 0.84 | 0.36 | 7 | 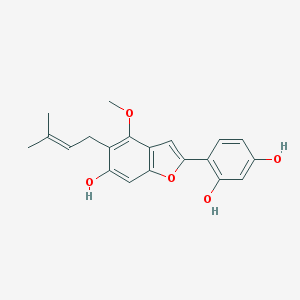 | licorice |
| MOL080 | Licoisoflavone | 41.61 | -0.27 | 0.37 | 0.42 | 19 | 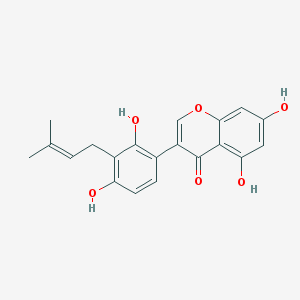 | licorice |
| MOL081 | Licoisoflavone B | 38.93 | -0.18 | 0.46 | 0.55 | 17 | 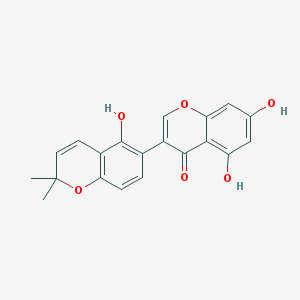 | licorice |
| MOL082 | licoisoflavanone | 52.47 | -0.22 | 0.39 | 0.54 | 20 | 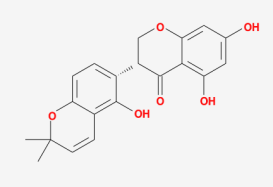 | licorice |
| MOL083 | shinpterocarpin | 80.3 | 0.68 | 1.1 | 0.73 | 30 | 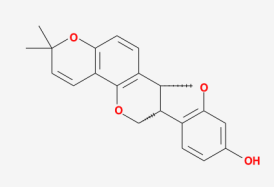 | licorice |
| MOL084 | (E)-3-[3,4-dihydroxy-5-(3-methylbut-2-enyl)phenyl]-1-(2,4-dihydroxyphenyl)prop-2-en-1-one | 46.27 | -0.4 | 0.41 | 0.31 | 12 | 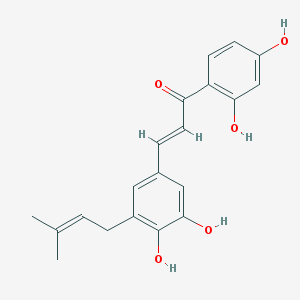 | licorice |
| MOL085 | liquiritin | 65.69 | -1.93 | -1.06 | 0.74 | 6 | 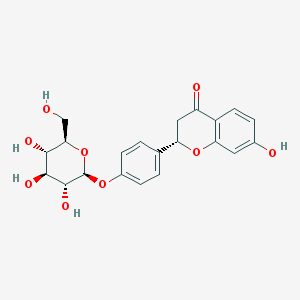 | licorice |
| MOL086 | licopyranocoumarin | 80.36 | -0.62 | 0.13 | 0.65 | 16 | 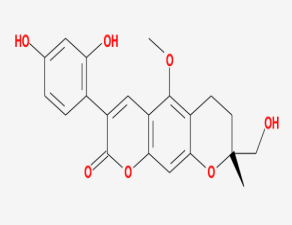 | licorice |
| MOL087 | Glyzaglabrin | 61.07 | -0.2 | 0.34 | 0.35 | 18 | 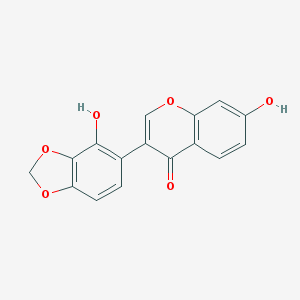 | licorice |
| MOL088 | Glabridin | 53.25 | 0.36 | 0.97 | 0.47 | 25 | 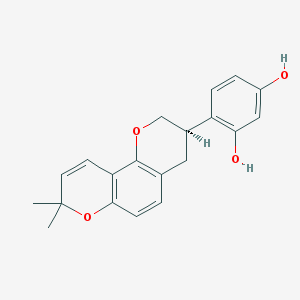 | licorice |
| MOL089 | Glabranin | 52.9 | 0.31 | 0.97 | 0.31 | 11 | 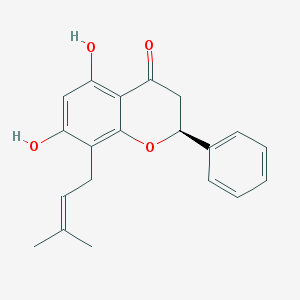 | licorice |
| MOL090 | Glabrene | 46.27 | 0.04 | 0.99 | 0.44 | 19 | 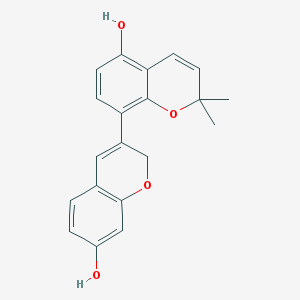 | licorice |
| MOL091 | Glabrone | 52.51 | -0.11 | 0.59 | 0.5 | 21 | 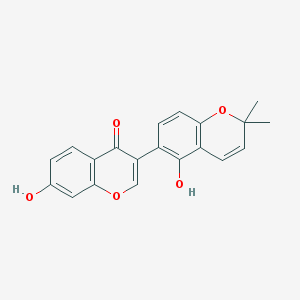 | licorice |
| MOL092 | 1,3-dihydroxy-9-methoxy-6-benzofurano[3,2-c]chromenone | 48.14 | -0.19 | 0.48 | 0.43 | 10 | 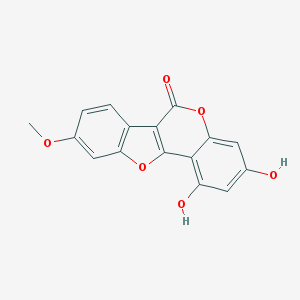 | licorice |
| MOL093 | 1,3-dihydroxy-8,9-dimethoxy-6-benzofurano[3,2-c]chromenone | 62.9 | -0.34 | 0.4 | 0.53 | 9 | 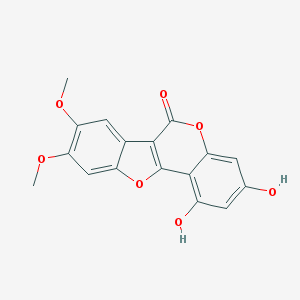 | licorice |
| MOL094 | Eurycarpin A | 43.28 | -0.06 | 0.43 | 0.37 | 19 | 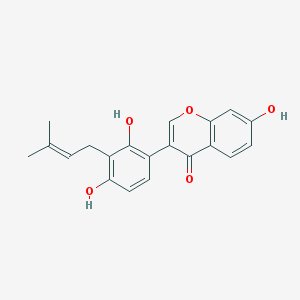 | licorice |
| MOL095 | (-)-Medicocarpin | 40.99 | -1.34 | -0.6 | 0.95 | 2 | 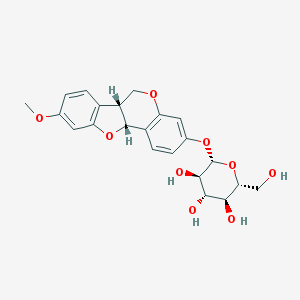 | licorice |
| MOL096 | Sigmoidin-B | 34.88 | -0.41 | 0.42 | 0.41 | 6 | 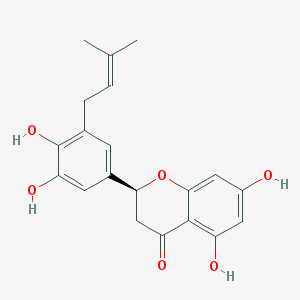 | licorice |
| MOL097 | (2R)-7-hydroxy-2-(4-hydroxyphenyl)chroman-4-one | 71.12 | -0.25 | 0.41 | 0.18 | 15 | 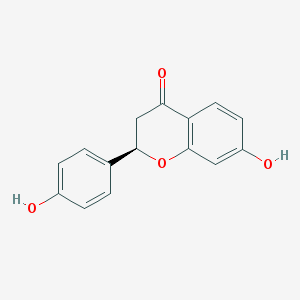 | licorice |
| MOL098 | (2S)-7-hydroxy-2-(4-hydroxyphenyl)-8-(3-methylbut-2-enyl)chroman-4-one | 36.57 | -0.04 | 0.72 | 0.32 | 12 | 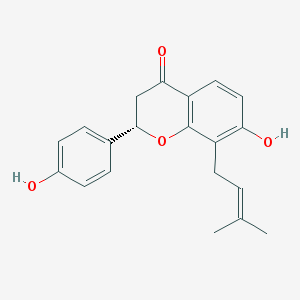 | licorice |
| MOL099 | Isoglycyrol | 44.7 | 0.05 | 0.91 | 0.84 | 7 | 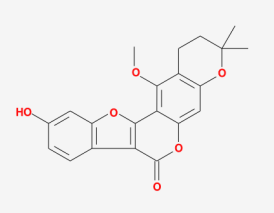 | licorice |
| MOL100 | Isolicoflavonol | 45.17 | -0.42 | 0.54 | 0.42 | 15 | 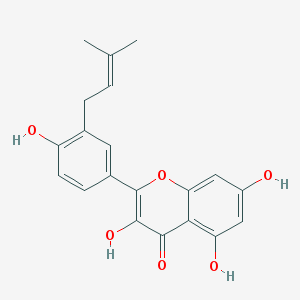 | licorice |
| MOL101 | Isoformononetin | 38.37 | 0.25 | 0.79 | 0.21 | 27 | 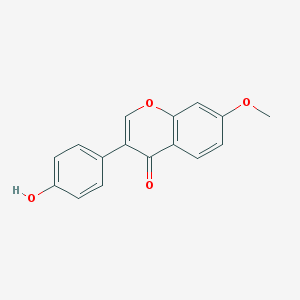 | licorice |
| MOL102 | 1-Methoxyphaseollidin | 69.98 | 0.48 | 1.01 | 0.64 | 29 |  | licorice |
| MOL103 | Quercetin der. | 46.45 | -0.44 | 0.39 | 0.33 | 17 |  | licorice |
| MOL104 | 3'-Hydroxy-4'-O-Methylglabridin | 43.71 | 0.73 | 1 | 0.57 | 28 |  | licorice |
| MOL105 | licochalcone a | 40.79 | -0.21 | 0.82 | 0.29 | 32 |  | licorice |
| MOL106 | 3'-Methoxyglabridin | 46.16 | 0.47 | 0.94 | 0.57 | 28 |  | licorice |
| MOL107 | 2-[(3R)-8,8-dimethyl-3,4-dihydro-2H-pyrano[6,5-f]chromen-3-yl]-licorice5-methoxyphenol | 36.21 | 0.61 | 1.12 | 0.52 | 31 |  | licorice |
| MOL108 | Inflacoumarin A | 39.71 | -0.24 | 0.73 | 0.33 | 15 |  | licorice |
| MOL109 | icos-5-enoic acid | 30.7 | 1.09 | 1.22 | 0.2 | 1 |  | licorice |
| MOL110 | Kanzonol F | 32.47 | 0.56 | 1.18 | 0.89 | 8 |  | licorice |
| MOL111 | 6-prenylated eriodictyol | 39.22 | -0.29 | 0.4 | 0.41 | 8 |  | licorice |
| MOL112 | 7,2',4'-trihydroxy－5-methoxy-3－arylcoumarin | 83.71 | -0.59 | 0.24 | 0.27 | 15 |  | licorice |
| MOL113 | 7-Acetoxy-2-methylisoflavone | 38.92 | 0.16 | 0.74 | 0.26 | 25 |  | licorice |
| MOL114 | 8-prenylated eriodictyol | 53.79 | -0.44 | 0.43 | 0.4 | 8 |  | licorice |
| MOL115 | gadelaidic acid | 30.7 | 0.94 | 1.2 | 0.2 | 1 |  | licorice |
| MOL116 | Vestitol | 74.66 | 0.3 | 0.86 | 0.21 | 30 |  | licorice |
| MOL117 | Gancaonin G | 60.44 | 0.23 | 0.78 | 0.39 | 20 |  | licorice |
| MOL118 | Gancaonin H | 50.1 | -0.14 | 0.6 | 0.78 | 12 |  | licorice |
| MOL119 | Licoagrocarpin | 58.81 | 0.61 | 1.23 | 0.58 | 29 |  | licorice |
| MOL120 | Glyasperins M | 72.67 | -0.04 | 0.49 | 0.59 | 26 |  | licorice |
| MOL121 | Glycyrrhiza flavonol A | 41.28 | -0.81 | -0.09 | 0.6 | 17 |  | licorice |
| MOL122 | Licoagroisoflavone | 57.28 | 0.09 | 0.71 | 0.49 | 18 |  | licorice |
| MOL123 | Odoratin | 49.95 | -0.24 | 0.42 | 0.3 | 20 |  | licorice |
| MOL124 | Phaseol | 78.77 | -0.06 | 0.76 | 0.58 | 14 |  | licorice |
| MOL125 | Xambioona | 54.85 | 0.52 | 1.09 | 0.87 | 8 |  | licorice |
| MOL126 | dehydroglyasperins C | 53.82 | -0.12 | 0.68 | 0.37 | 18 |  | licorice |
| MOL127 | quercetin | 46.43 | -0.77 | 0.05 | 0.28 | 152 |  | licorice |
